# First interchromosomal insertion in a patient with cerebral and spinal cavernous malformations

**DOI:** 10.1038/s41598-020-63337-5

**Published:** 2020-04-14

**Authors:** Robin A. Pilz, Konrad Schwefel, Anja Weise, Thomas Liehr, Philipp Demmer, Andreas Spuler, Stefanie Spiegler, Eberhard Gilberg, Christian A. Hübner, Ute Felbor, Matthias Rath

**Affiliations:** 1 Department of Human Genetics, University Medicine Greifswald, and Interfaculty Institute of Genetics and Functional Genomics, University of Greifswald, Greifswald, Germany; 2 Institute of Human Genetics, Jena University Hospital, Friedrich Schiller University, Jena, Germany; 3 Institute of Medical Diagnostics, IMD Potsdam, Potsdam, Germany; 4 Department of Neurosurgery, Helios Hospital Berlin Buch, Berlin, Germany

**Keywords:** Clinical genetics, Genetics of the nervous system, Structural variation, Cerebrovascular disorders, Neurology

## Abstract

Autosomal dominant cerebral cavernous malformations (CCM) are leaky vascular lesions that can cause epileptic seizures and stroke-like symptoms. Germline mutations in either *CCM1*, *CCM2* or *CCM3* are found in the majority of patients with multiple CCMs or a positive family history. Recently, the first copy number neutral inversion in *CCM2* has been identified by whole genome sequencing in an apparently mutation-negative CCM family. We here asked the question whether further structural genomic rearrangements can be detected within NGS gene panel data of unsolved CCM cases. Hybrid capture NGS data of eight index patients without a pathogenic single nucleotide, indel or copy number variant were analyzed using two bioinformatics pipelines. In a 58-year-old male with multiple CCMs in his brain and spinal cord, we identified a 294 kb insertion within the coding sequence of *CCM2*. Fine mapping of the breakpoints, molecular cytogenetic studies, and multiplex ligation-dependent probe amplification verified that the structural variation was an inverted unbalanced insertion that originated from 1p12-p11.2. As this rearrangement disrupts exon 6 of *CCM2* on 7p13, it was classified as pathogenic. Our study demonstrates that efforts to detect structural variations in known disease genes increase the diagnostic sensitivity of genetic analyses for well-defined Mendelian disorders.

## Introduction

Cerebral cavernous malformations (CCM; MIM: 116860, 603284, 603285) are irregular clusters of enlarged and thin-walled vessels that can present as sporadic or autosomal dominant cerebrovascular disease. Aside from epileptic seizures and headaches, CCM patients may present with stroke-like symptoms due to chronic or acute bleeding events^1^. Pathogenic germline variants have been identified in *CCM1* (also known as *KRIT1)*^2,3^, *CCM2*^4,5^, and *CCM3* (*PDCD10)*^6^. The mutational spectrum primarily includes nonsense, frameshift, splice, and copy number variants (CNVs). 282 unique *CCM1*, 84 *CCM2* and 75 *CCM3* variants are classified as disease-causing in the Human Gene Mutation Database (HGMD Professional 2019.3)^7^. Depending on the inclusion criteria for genetic analyses, mutation detection rates of 87 to 98% have been reported for familial CCM cases and up to 60% for sporadic ones^8–11^. The mutation detection rate in the latter group could even be higher if patients with associated developmental venous anomalies or a history of radiotherapy to the brain were excluded^1,12^. Although pathogenic variants in a yet unknown *CCM4* candidate gene have been discussed for unresolved cases^13^, we recently were able to identify the first copy number neutral inversion in *CCM2* in an apparently mutation-negative CCM family by whole genome sequencing (WGS)^14^.

Disease-causing structural variants (SVs) may explain a part of the “missing heritability” in rare diseases^15^. SVs are defined as structural and quantitative chromosomal rearrangements that compromise cytogenetically visible and submicroscopic variants^16,17^. They contribute to phenotypic variation but can also cause human disease^17^. In fact, rare SVs are even more likely to be deleterious than rare single nucleotide variants (SNVs)^18,19^. Deletions, duplications, insertions, translocations, and inversions may directly disrupt the organization of a disease gene or affect its transcriptional regulation by positional effects^16,17^. Using a multi-platform WGS approach, Chaisson and colleagues have demonstrated in 2019 that more than 27,000 SVs (≥50 bp) can be found per human genome^20^. In contrast, an individual human genome harbors approximately 4,000,000 to 5,000,000 SNVs and up to 800,000 indels, which are defined as insertions or deletions with a length of up to 49 bp^18^. However, the identification of SVs remains much more challenging than SNV or indel calling in a diagnostic setting. While deletions and duplications of exonic sequences can be reliably detected with qPCR, multiplex ligation-dependent probe amplification or NGS-based CNV detection algorithms, copy number neutral SVs or deletions and duplications in non-coding regions of known disease-associated genes may escape targeted genetic approaches.

In this study, we have analyzed the hybrid capture NGS gene panel data of eight genetically unresolved CCM index patients for the presence of SVs in *CCM1*, *CCM2* or *CCM3*. An interchromosomal insertion which led to an interruption of exon 6 of the *CCM2* gene was identified in a sporadic CCM patient.

## Results

### Clinical findings

**Figure 1:**
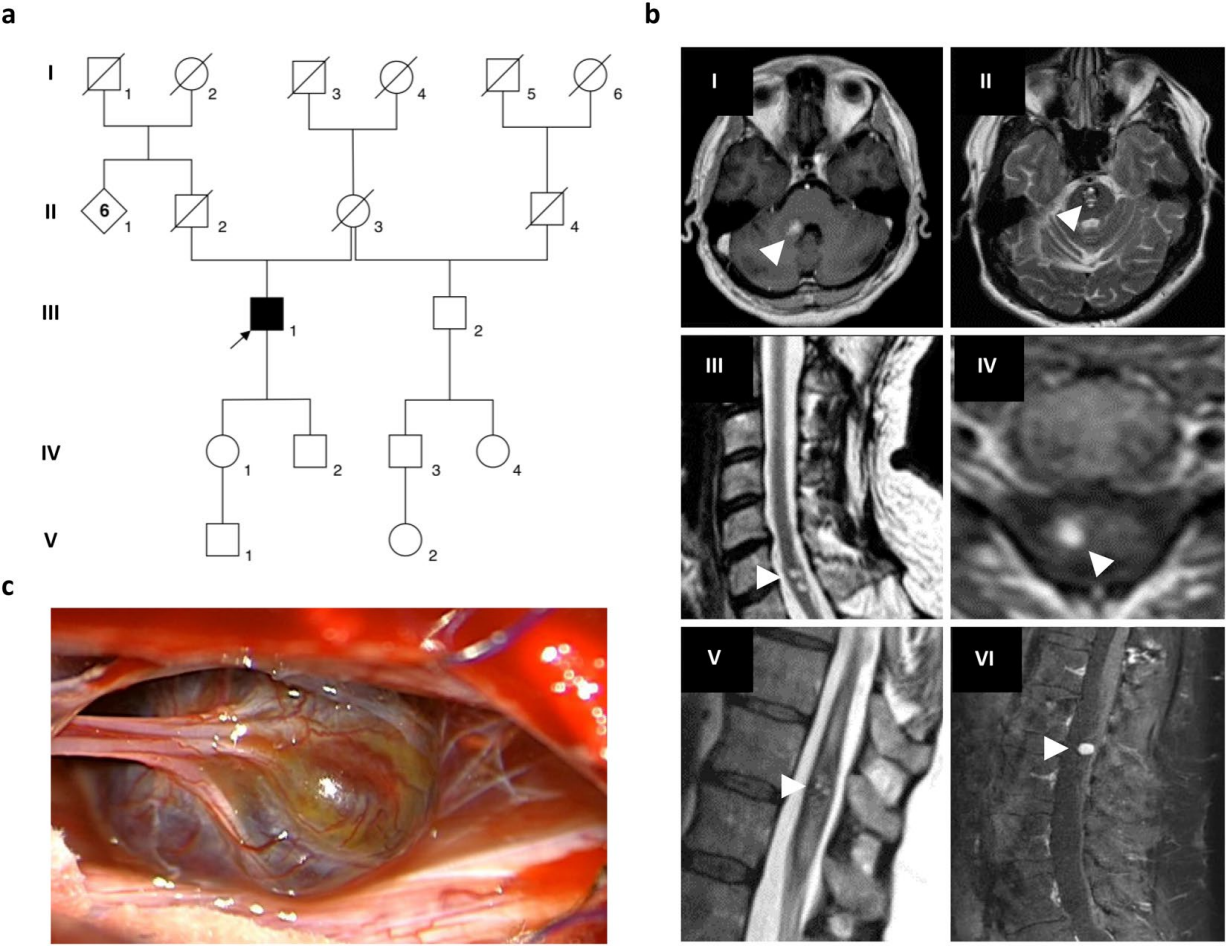
Multiple cerebral and spinal cavernomas in a sporadic CCM patient. (**a**) Pedigree of the CCM index case from pedigree 1 (III:1, arrow). (**b**) Repetitive magnetic resonance imaging (MRI) of the index patient’s brain and spinal cord showing progression of CCM disease with two cavernous malformations in the pons (I, II), intramedullary lesions in the cervical (III, IV) and thoracic spinal cord (V) as well as a CCM in the cauda equina (VI). MRI images were acquired between 2005 and 2019. White arrowheads indicate CCMs. (**c**) Intraoperative photograph of the cauda equina cavernous malformation.

The male index patient III:1 (pedigree 1, Fig. 1a) was referred to genetic counselling at the age of 58 years because of multiple symptomatic CCMs. Magnetic resonance imaging (MRI) documented at least nine cavernous lesions with surrounding hemosiderin deposits in his brain and spinal cord (Fig. 1b). One brainstem cavernoma and three CCMs in his thoracic, cervical, and lumbar spine had already been resected because of acute bleeding events at the age of 44, 50, and 58, respectively (Fig. 1c, Supplementary Video S1). The index patient also reported an atrial septal defect and a traumatic L1 vertebral body fracture at the age of 55 years which had been treated with balloon kyphoplasty. On physical examination, he presented a partial loss of vibration and fine touch sensation below the level of T10 and impaired sensation of temperature on the contralateral side. In addition, he had an ataxic gait with right foot drop. The index patient’s father (II:2) died of gastric cancer when he was 72 years old. Cavernous lesions or neurological symptoms that would be suggestive for CCM had not been documented for him. The mother of the index patient (II:3) passed away at the age of 90 years with no signs of CCM either. Both children (IV:1 and IV:2) are healthy, and there were no other relatives who had CCMs or reported epileptic seizures, stroke-like symptoms or chronic headaches.

### Identification of an interchromosomal insertion

**Figure 2:**
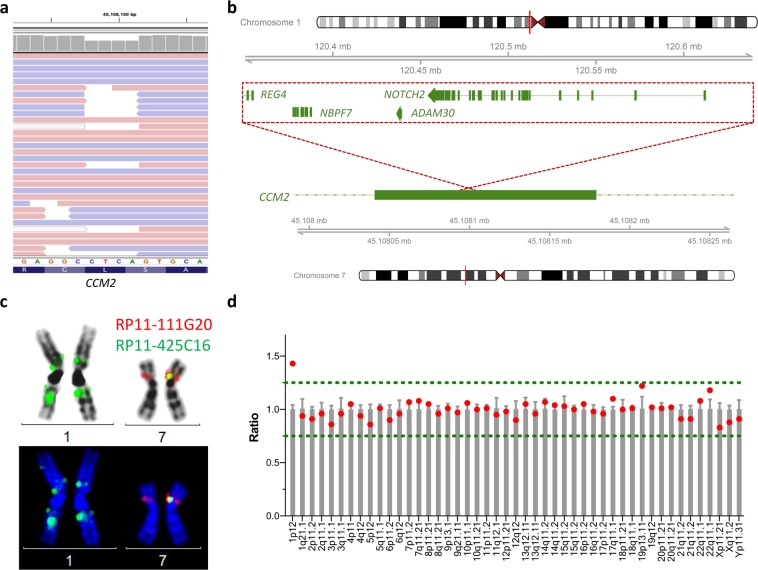
Identification of an unbalanced interchromosomal insertion in *CCM2*. (**a**) Read alignment of the hybrid capture NGS data of III:1. Shown is a part of exon 6 of the *CCM2* gene. The coverage plot indicated significantly reduced read depths at positions [hg19] chr7:45,108,098-45,108,101 but no reads were found that covered a deletion of 4 bp. (**b**) Schematic depiction of the identified interchromosomal insertion. Material from 1p12-p11.2 was found as an inverted insertion in exon 6 of the *CCM2* gene on 7p13. (**c**) Verification of the chromosomal rearrangement [46,XY.ish ins(7;1)(p13;1p11)(RP11-425C16+,RP11-111G2 +)] by fluorescence *in situ* hybridization. There is a known crosshybridization of the applied probe RP11–425C16 in 1q21.1, which does not need to be considered further taking into account all other data presented here. (**d**) MLPA analysis of the centromere region of chromosome 1 indicated three copies of the *NOTCH2* gene (1p12) which is part of the inserted fragment.

Gene panel sequencing of *CCM1*, *CCM2*, and *CCM3* for index patient III:1 (Fig. 1) and routine bioinformatics analyses identified no pathogenic SNV, indel or CNV. However, a high number of split reads in *CCM2* was noticed in NGS gene panel data of this proband. These deviant reads could be grouped into two clusters based on their mapping. Reads of the first group mapped with one part to the 5' half of exon 6 of *CCM2*, which is located on 7p13, and with the other part to 1p11.2 or 1q21.1. Due to known sequence homologies, aberrant mapping on chromosome 1 could not be further specified. Split reads of the second group partially mapped to the 3' half of *CCM2* exon 6 but also to 1p12. Notably, detection of deviant reads required the SeqNext module of the Sequence Pilot tool for mapping and alignment. Using the mapping and alignment information of the MiSeq Reporter Software resulted in significantly reduced read depth at positions [hg19] chr7:45,108,098-45,108,101. However, split reads were filtered out by SeqNext and no variant was called since there were no reads that covered a heterozygous deletion of these four nucleotides (Fig. 2a).

Based on the NGS data of index patient III:1, we suspected an interchromosomal insertion from 1p12-p11.2 into the coding region of *CCM2* on 7p13 (Fig. 2b). This rearrangement was finally made visible by fluorescence *in situ* hybridization (FISH) on metaphase chromosomes from cultured blood lymphocytes of the index patient (Fig. 2c). The inserted fragment covers a genomic region of 294 kb that contains the complete *NOTCH2* (MIM: 600275), *ADAM30* (604779), and *NBPF7* (613997) genes as well as parts of *REG4* (609846). MLPA analysis with a centromere kit further indicated the presence of three copies of *NOTCH2* which is the only gene in the inserted fragment with an associated phenotype (Fig. 2d). Loss-of-function mutations and pathogenic missense variants in this gene have been reported in patients with Alagille (MIM: 610205) and Hajdu-Cheney syndrome (102500). However, the index patient did not meet the clinical diagnostic criteria of either syndrome.

### Fine mapping of the breakpoints

**Figure 3:**
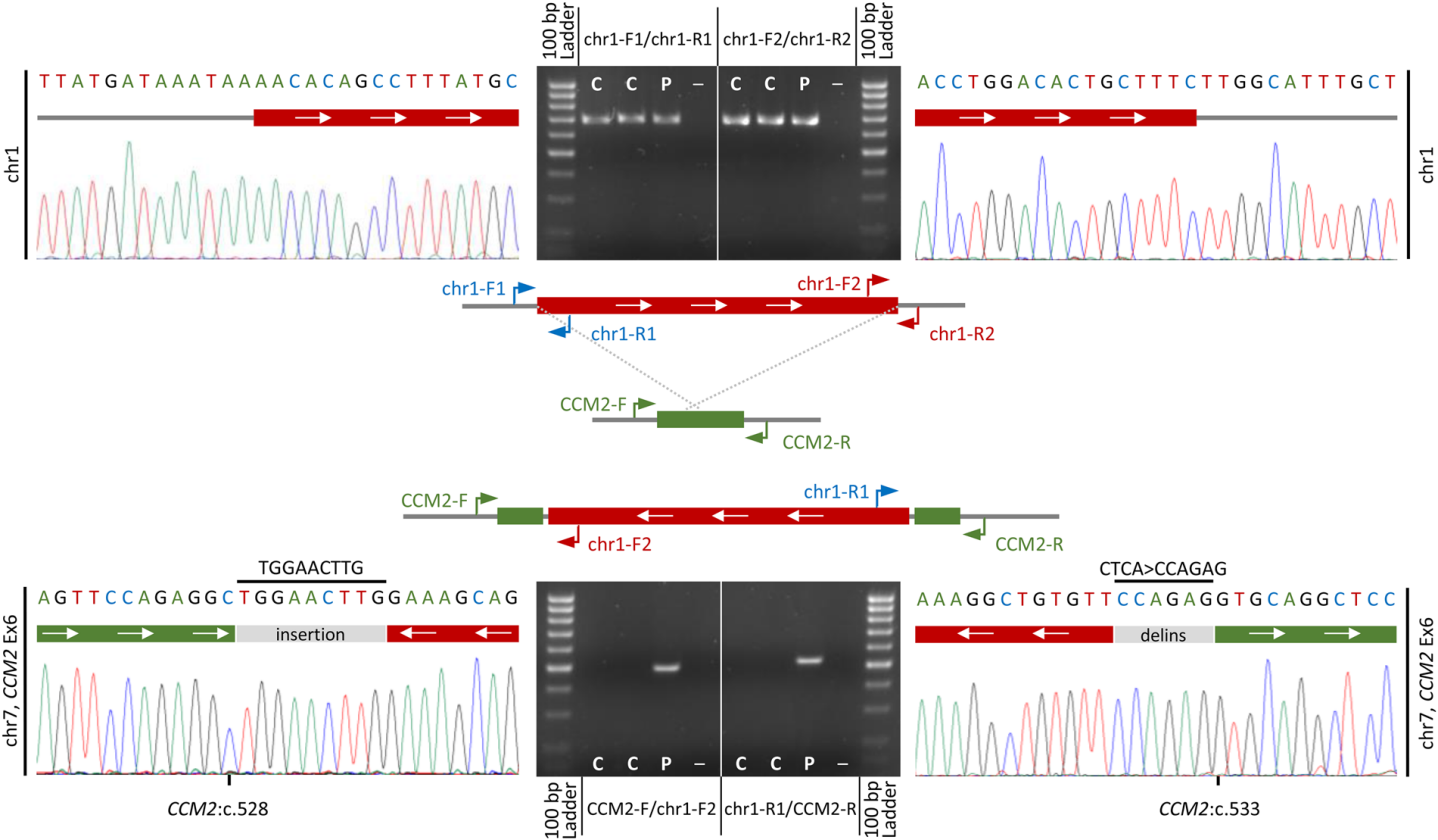
Fine mapping of the breakpoints by Sanger sequencing. Scheme of the normal and inverted insertion alleles (middle panel). PCR and sequencing primers (chr1: chr1-F1 + R1 and chr1-F2 + R2; chr7: CCM2-F + R) are depicted as blue, red, and green arrows, respectively. PCR products and chromatograms of the normal allele on chr1 (chr1-F1/R1 and chr1-F2/R2) are depicted in the upper panel. PCR products and chromatograms of the inverted insertion on chr7 (CCM2-F/chr1-F2 and chr1-R1/CCM2-R) are depicted in the lower panel. Fine mapping also revealed an additional small insertion and an indel variant at the breakpoints. C = healthy control; P = index patient; – = negative control; Ex = exon.

We next amplified the breakpoints of the interchromosomal insertion for its precise molecular characterization. Sanger sequencing allowed us to fine map the breakpoints to [hg19] chr1:120,347,265, chr1:120,641,440, and chr7:45,108,098–45,108,101, respectively. It also verified that the fragment of 1p12-p11.2 was inserted in an inverted orientation on 7p13 and flanked by a small insertion and a deletion-insertion variant in which the four base pairs with reduced sequencing depths were replaced by six nucleotides of unknown origin (Fig. 3). While the breakpoint on 1p11.2 locates within a long interspersed nuclear element (LINE), no repeat elements were found in close proximity to the breakpoints on 1p12 or 7p13. As the heterozygous SV interrupts exon 6 of the *CCM2* gene, it was classified as pathogenic for CCM. In summary, the results of our molecular, cytogenetic, and molecular cytogenetic studies demonstrated that index patient III:1 is a heterozygous carrier of a submicroscopic, unbalanced, interchromosomal, and inverted insertion.

### Screening for SVs in mutation-negative CCM patients

We finally wanted to know if SVs can also be identified in the hybrid capture NGS data of seven additional CCM patients without any pathogenic SNV, indel or CNV in *CCM1*, *CCM2* or *CCM3* that had been analyzed between 2017 and 2019 (Supplementary Table S1). Therefore, we chose the Agilent SureCall tool, which allowed us to search for translocation events based on the presence of split reads in their NGS data. The index patient III:1 from family 1 was used as a positive control. The tool precisely re-identified his SV but did not detect any further chromosomal rearrangements in the other CCM cases. Furthermore, no split reads were found by visual inspection of their NGS data.

## Discussion

In this study, we have identified the first interchromosomal insertion, sometimes also referred to as interchromosomal insertional translocation (IT), in a CCM patient. Notably, current genetic analysis cannot clarify the molecular basis of CCM disease in 2 to 13% of familial cases and up to 40% of sporadic CCM patients^1^. Besides phenocopies, somatic mosaicism and pathogenic variants in an unknown *CCM4* gene or non-coding regions of *CCM1*, *CCM2* or *CCM3* have been discussed as possible explanations for these patients^21–23^. However, the identification of an interchromosomal insertion in one of eight unsolved CCM cases and the copy number neutral inversion in *CCM2*, that we have reported recently^14^, demonstrate that SVs also need to be considered. It is remarkable that both rearrangements identified so far have been detected in *CCM2* which is the largest of the three CCM genes. However, future studies will have to show whether *CCM2* is more susceptible to the occurrence of SVs or whether further genomic rearrangements can be detected in *CCM1* and *CCM3* with more sensitive techniques.

ITs and other SVs have a higher prevalence than previously thought^20,24,25^. FISH confirmation and parental follow-up studies of CNVs that had been identified by clinical array comparative genomic hybridization (aCGH) analysis revealed that ITs can be found in approximately 1 of 500 patients referred to aCGH analysis^24^. This observation suggests that their prevalence might also be underestimated in patients with CCM or other inherited disorders for whom aCGH is not part of standard genetic analyses. Sophisticated bioinformatics algorithms have been developed to identify SVs from short-read NGS data based on inconsistent paired-end mapping, the presence of split reads or changes in read depth^26,27^. While CNVs can be efficiently detected with NGS-based approaches, inversions and ITs that do not cause gain or loss in the number of *CCM1*, *CCM2* or *CCM3* alleles might be regularly missed with targeted gene panel sequencing. Furthermore, the analytical sensitivity of the available bioinformatics tools is still incomplete. Notably, only one of the two tools that were used for mapping and alignment in our current study allowed us to detect aberrant reads in the NGS data analysis for the index patient from pedigree 1.

The search for chromosomal rearrangements is becoming more and more important in genetic research projects that try to solve undiagnosed cases with rare diseases. While the detection of ITs within the coding region of a gene seems to be relatively straightforward with special bioinformatics tools and filter criteria, insertions into intronic, promotor or cis-regulatory regions that can also impair gene regulation^16^ can hardly ever be detected with PCR- or hybrid capture-based target enrichment strategies and short-read sequencing. Recently, short-read WGS and a new bioinformatics pipeline revealed disease-causing SVs in 16 out of 477 patients of the Undiagnosed Diseases Network (UDN)^28^. However, third-generation sequencing technologies will probably further improve the diagnostic sensitivity for the identification of chromosomal rearrangements as the probability of spanning the whole SV and the mappability in repetitive regions are higher for long reads^29,30^. In a cohort of 1,324 undiagnosed probands with rare diseases from the NIHR BioResource research study, three cases with a complex pathogenic SV were identified by short-read WGS and a duplication-inversion-duplication event of unknown clinical significance was resolved in another proband by long-read sequencing^31^. Whole genome long-read sequencing also identified a heterozygous ~2.2 kb deletion in *PRKAR1A* which is a known disease gene for autosomal dominant Carney complex in a patient with multiple tumours for whom initial targeted *PRKAR1A* sequencing and short-read WGS had not revealed any pathogenic variant^32^. Several other examples illustrate that long-read sequencing enables effective SV calling and could help to solve undiagnosed cases^33^. Therefore, long-read WGS is a promising next step for the yet unsolved CCM cases of our cohort. These have already been checked for CNVs in *CCM1*, *CCM2*, and *CCM3* but third-generation sequencing technologies would allow the detection of CNVs and copy number neutral SVs in a genome-wide approach. Nevertheless, the combination of multiple sequencing technologies might be still necessary to achieve sufficient insertion and inversion detection sensitivities^20^.

The identification of a pathogenic SNV, indel, CNV or SV is essential for genetic counselling of CCM families and always raises the question whether it is an inherited or a *de novo* variant. As both parents of the index patient from pedigree 1 died without CCMs, epileptic seizures or stroke-like symptoms, one might speculate that his SV is a *de novo* mutation. In line with this hypothesis, a recent analysis of the Genome Aggregation Database (gnomAD) Consortium suggested a *de novo* mutation rate of 0.35 SVs per generation (95% confidence interval: 0.18–0.52)^25^. Given that a significant number of SVs might have been missed with short-read WGS, this projection likely underestimates the real number of *de novo* SVs per genome. However, we can also not exclude a cryptic inherited case in pedigree 1 since the penetrance of CCM is incomplete and up to 45% of *CCM2* mutation carriers remain asymptomatic^9^. Indeed, Nowakowska and colleagues demonstrated that a significant number of apparently *de novo*, interstitial CNVs that had been found in patients with multiple congenital anomalies or mental retardation were actually the result of an unbalanced transmission of a derivative chromosome from one parent with a balanced insertional translocation^34^. Furthermore, the transmission from an obviously unaffected parent that carries the same unbalanced rearrangement as its affected child has also been reported^24^. Unfortunately, DNA samples of the index patient’s parents were not available and there were no other affected family members to address the origin of the chromosomal rearrangement or to demonstrate co-segregation of the SV and CCM disease.

In conclusion, our study adds the first interchromosomal insertion to the *CCM* mutation spectrum and suggests that hard-to-detect SVs might account for more CCM cases than previously thought. Together with other literature reports of copy number neutral chromosomal rearrangements in known disease genes^14,35,36^, our results support the hypothesis that efforts to detect SVs might be more promising than the search for novel candidate genes for well-defined Mendelian disorders.

## Methods

### Study population and ethical considerations

Genomic DNA was isolated from peripheral blood lymphocytes of all study participants with written informed consent using the NucleoSpin Blood L Kit according to the manufacturer’s instructions (Macherey-Nagel, Düren, Germany). A Qubit 2.0 Fluorometer (Thermo Fisher Scientific, Waltham, USA) was used to measure DNA concentrations before NGS target enrichment. DNA purity was determined on a NanoPhotometer instrument (Implen, München, Germany). All procedures performed in this study involving human participants were in accordance with the 1964 Helsinki declaration and its later amendments.The study protocol was approved by the local ethics committee of the University Medicine Greifswald (BB 047/14) and written informed consent was obtained from index patient III:1 to publish the medical information and images that are presented here.

### Target enrichment, next-generation sequencing, and bioinformatics analyses

All exons (±20 bp) of *CCM1* (Locus Reference Genomic sequence: LRG_650t1), *CCM2* (LRG_664t2), and *CCM3* (LRG_651t1) were defined as target regions for NGS gene panel analysis. A Nextera Rapid Capture Custom Enrichment Kit (Panel ID: 113402; Illumina, San Diego, USA) or an Agilent SureSelect custom library (Panel ID: 3152261, Agilent Technologies, Santa Clara, USA) and a SureSelect Reagent Kit (Agilent Technologies) were used for target enrichment and library preparation according to the manufacturers’ instructions, respectively. Pre- and post-capture libraries were analyzed on a 2100 Bioanalyzer instrument (Agilent Technologies). Indexed libraries were pooled and sequenced on a MiSeq instrument (Illumina) as 2 × 150 bp paired-read runs. The MiSeq Reporter Software (Illumina) was used for demultiplexing and FASTQ file generation. Mapping and alignment were independently performed for each sample with the MiSeq Reporter Software and the SeqNext module of the Sequence Pilot software (JSI medical systems, Ettenheim, Germany). The SeqNext module was also used in a read depth-based approach to identify CNVs in *CCM1*, *CCM2*, and *CCM3* as described previously^37^. BAM files that had been generated with the MiSeq Reporter Software were further analyzed for translocation events with the SureCall 4.1.1.5 software (Agilent Technologies), and aligned reads were grouped by the mate chromosome in the Integrative Genomics Viewer^38^.

### Sanger sequencing and multiplex ligation-dependent probe amplification

Specific primer pairs were designed to amplify the suspected breakpoints of a novel interchromosomal insertion identified in *CCM2*. Primer sequences are available upon request. PCR products were purified with the ExoSAP-IT Cleanup Reagent (Thermo Fisher Scientific). Sanger sequencing was performed on a SeqStudio Genetic Analyzer (Thermo Fisher Scientific) following established protocols. The SALSA MLPA P181-B1 Centromere mix 1 (MRC-Holland, Amsterdam, The Netherlands) was used according to the manufacturer’s instructions to determine copy number variations of a part of the *NOTCH2* gene (MIM: 600275) which is located on chromosome 1p12 (hg19: 120,454,176–120,612,317).

### Chromosome analysis and molecular cytogenetic studies

Karyotyping was performed following standard procedures on metaphases obtained from PHA stimulated blood lymphocytes. Fluorescence *in situ* hybridization (FISH) was used to confirm the interchromosomal insertion using standard protocols. The following BAC clones were used: RP11–425C16 in 1p12 (hg19: 120,176,963–120,358,983) and RP11–111G20 in 7p12.3~13 (hg19: 45,283,437–45,448,789).

### Ethical approval

All procedures performed in this study involving human participants were in accordance with the ethical standards of the institutional and/or national research committee (University Medicine Greifswald; BB 047/14) and with the 1964 Helsinki declaration and its later amendments or comparable ethical standards.

## Supplementary information

Supplementary Information.

Supplementary Information2.

## Data Availability

All relevant data generated or analysed during this study are included in this published article and its supplementary information files.
