# Supplementary material for: First interchromosomal insertion in a patient with cerebral and spinal cavernous malformations
Source: Sci Rep. 2020 Apr 14;10:6306. doi: 10.1038/s41598-020-63337-5 (PMC7156631; doi:10.1038/s41598-020-63337-5)
Supplement: Supplementary file 1 — Supplementary Information. [file 41598_2020_63337_MOESM1_ESM.pdf]

SUPPLEMENTARY MATERIAL

First interchromosomal insertion in a patient with cerebral and spinal cavernous malformations

Authors:

Robin A. Pilz[1], Konrad Schwefel[1], Anja Weise[2], Thomas Liehr[2], Philipp Demmer[3], Andreas Spuler[4], Stefanie Spiegler[1], Eberhard Gilberg[1], Christian A. Hübner[2], Ute Felbor[1], Matthias Rath[1,*]

Affiliations:

[1] Department of Human Genetics, University Medicine Greifswald, and Interfaculty Institute of Genetics and Functional Genomics, University of Greifswald, Greifswald, Germany.

[2] Institute of Human Genetics, Jena University Hospital, Friedrich Schiller University, Jena, Germany.

[3] Institute of Medical Diagnostics, IMD Potsdam, Potsdam, Germany.

[4] Department of Neurosurgery, Helios Hospital Berlin Buch, Berlin, Germany.

***Correspondence:**

Matthias Rath, MD

Department of Human Genetics, University Medicine Greifswald, Fleischmannstraße 43,

D 17475 Greifswald, Germany.

Email: matthias.rath@med.uni-greifswald.de

Supplementary Table S1 Clinical characteristics of the index patient from family 1 and seven additional CCM patients analyzed in this study.

	**N = 8**
Mean age at genetic analysis ± standard deviation in years (range)	40.0 ± 19.4<br>(7 - 63)
Sex (male : female)	2 : 6
Family history[a] (familial : sporadic)	1 : 7

[a] One CCM index patient was classified as familial case based on the presence of family members with clinical symptoms that were suggestive of CCM disease (seizures, hemorrhagic stroke).
